# Recognizing anxiety and depression in cancer patients based on speech and facial expressions

**DOI:** 10.3389/fpsyt.2026.1813727

**Published:** 2026-05-14

**Authors:** Na Xu, Tao Wu, Li Sun, Ping Xin, Xu Tan, Na Luo, Yi Liu

**Affiliations:** 1 Union Hospital, Tongji Medical College, Huazhong University of Science and Technology, Wuhan, China; 2 School of Nursing, Tongji Medical College of Huazhong University of Science and Technology, Wuhan, China; 3 Department of Executive Management,Shenzhen Guoguan Intelligent Technology Co., Ltd., Shenzhen, China

**Keywords:** affective computing, anxiety detection, computational paralinguistics, depression detection, multimodal fusion, neural networks

## Abstract

**Purpose:**

To address the anxiety and depression experienced by cancer patients due to the stress of diagnosis and treatment, as well as the limitations of traditional assessment methods characterized by high subjectivity and low efficiency, this study aims to develop a multimodal fusion approach for the simultaneous and precise evaluation of these two psychological states.

**Patients and methods:**

A speech-video dataset of clinically diagnosed cancer patients was used. This study proposes a multimodal fusion approach: for depression recognition, We employ the HuBERT pre-training architecture based on Transformers, integrating specific acoustic features of depression with textual content to achieve accurate classification of depression through a voice-text modality. For anxiety recognition, a multi-task convolutional neural network is designed to infer anxiety status from the facial expressions.

**Results:**

Experiments conducted on a speech-video dataset of clinically diagnosed cancer patients demonstrates that the multimodal fusion model achieves a depression recognition F1 value of 0.85 and an anxiety recognition F1 value of 0.74, significantly outperforming the unimodal model.

**Conclusion:**

The results of the two modalities are fused by decision-level weighted averaging to realize the simultaneous assessment of anxiety and depression in cancer patients. The study may provide technical support for rapid, noninvasive screening of psychological status in cancer patients.

## 1 Introduction

Data published in *The Lancet* indicates that “The impact of cancer in China remains severe. Although the incidence of cancer in China is comparable to the global average, mortality rates are significantly higher. The country ranks first in the world in terms of the number of cancer deaths per year (1).” The physical and psychological challenges imposed by cancer and its treatments not only diminish patients’ quality of life and hope for survival, but also subject them to physical pain and uncertainty regarding disease progression, often triggering complex emotional disturbances (2, 3). A study has shown that depression is more prevalent among patients with chronic diseases and fatal diseases such as cancer than in healthy population (4). The global summary prevalence of depression among cancer survivors was 33.16% (95% CI 27.59-38.74), while anxiety had a prevalence of 30.55% (95% CI 24.04-37.06) (5). Notably, studies have confirmed that even mild depression and anxiety can substantially reduce the quality of life of cancer patients (6). In fact, depression and anxiety are very common psychological disturbances in the cancer patient population, and they often trigger a series of adverse physical and psychological reactions, such as increased physical pain, decreased treatment efficacy, and elevated mortality risk (7, 8). This phenomenon also highlights the importance of preventing and treating depression for cancer patients.

A meta-analysis (9) showed anxiety prevalence rates of 24.4% and depression prevalence rates of 23.7% among cancer patients. In routine oncological treatment settings, psychological distress, including mental disorders, is overlooked in 30% to 50% of patients (10). And undiagnosed and untreated anxiety and depression in cancer patients can negatively impact health outcomes and potentially increase cancer-related complications and mortality risk (11). In certain regions, social stigma and cultural perceptions surrounding cancer and mental health hinder patients from seeking psychological support (12). Current psychological assessments for cancer patients primarily rely on questionnaires and physician interviews. However, surgeons often lack the ability to accurately assess patients’ psychological states and may underestimate their distress (13). Patients often struggle to complete questionnaires due to physical weakness and cognitive overload. Additionally, symptoms related to anxiety and depression may be overlooked or concealed by patients (14). These factors collectively increase the difficulty of diagnosing their psychological issues. These limitations highlight the need for complementary, objective screening methods that can be integrated into routine care without increasing patient burden.

Recent advances in affective computing have opened new avenues for mental health screening by leveraging unstructured data such as speech, facial expressions, and free text (15–17). Compared with structured data such as scale scores and clinical diagnoses, unstructured data like free text, images, audio and video have richer dimensions and stronger dynamics, and can capture deep psychological and behavioral characteristics that are difficult to quantify by traditional methods (18, 19). For negative emotions such as depression and anxiety, the abnormal psychological states of patients are not merely manifested as subjective emotional experiences, but can be explicitly expressed through a series of non-verbal behavioral signals (17, 20, 21). Among them, depressive states are often accompanied by behavioral characteristics such as flat intonation, slowed speech rate, and reduced facial expression activity. Anxiety is often manifested as rapid speech and tense facial muscles, etc. (15, 17). Importantly, these vocal and facial signatures are partly automatic and less amenable to voluntary control than questionnaire responses, thereby functioning as behavioral and physiological markers that can capture latent distress even when patients consciously minimize or conceal symptoms. Leveraging speech and facial expressions as multimodal “digital biomarkers” may therefore help to circumvent stigma-driven under-reporting and provide a more objective, continuous window into the affective state of cancer patients.

Utilizing multimodal frameworks to process data encompassing multiple modalities (such as video, audio, text) can significantly enhance model performance (22). Although previous multimodal approaches have explored various combinations of audio, text, and video data for detecting mental disorders (23), studies based on Chinese cancer patient data remain scarce. Different modalities exhibit varying sensitivities to different emotional dimensions. Speech features are more indicative of depressive symptoms, such as slowed speech, reduced prosody, and emotional flatness. Facial dynamics, on the other hand, are more sensitive to anxiety-related arousal, tension, and facial expression suppression (24). Therefore, this study develops a multimodal framework for concurrent risk assessment of anxiety and depression among cancer patients. Audio recordings and corresponding textual transcripts are used to capture affective and linguistic features closely associated with depressive symptoms. Video recordings are analyzed to extract facial expression dynamics that reflect anxious arousal and emotional regulation. A decision fusion strategy is then employed to integrate complementary information across modalities, enabling joint prediction of depression and anxiety risks.

This study explicitly positions the developed system as a supportive screening tool for psycho-oncology care, with no intent to substitute clinical diagnosis or psychiatric evaluation. Its core objective is to construct and internally validate a multimodal fusion model. This model aims to identify individuals at elevated risk of clinically significant anxiety or depression per the predefined cutoff thresholds of standardized questionnaires(PHQ-9, GAD-7), thereby facilitating more comprehensive assessments by mental health professionals.

## 2 Methods

The framework for the audio-visual depression detection method comprises four development stages: collection and processing of datasets (Section 2.1), Voice-Text Modal Depression Recognition (Section 2.2), Facial Expression Anxiety Recognition (Section 2.3), and multimodal fusion strategy (Section 2.4).

### 2.1 Dataset collection and processing

The authors recruited cancer patients hospitalized in a tertiary care hospital in Wuhan, Hubei, China, from April 2024 to October 2025 and established a database. Eligibility criteria included: (1) histopathologically confirmed malignancy; (2) capacity to provide informed consent; and (3) ability to complete psychometric assessments. Exclusion criteria comprised: (1) documented cognitive impairment (MMSE score<24); (2) history of organic psychotic disorders; and (3) severe communication barriers. All patients gave informed consent and participated in this study voluntarily, and the study was approved by the Ethics Committee of the hospital (2024[0256]). After obtaining informed consent from the patient, the Patient Health Questionnaire-9 (PHQ-9) and Generalized Anxiety Disorder-7 (GAD-7) self-report questionnaires were completed. After completing the questionnaires, video and audio data were collected from patients according to their wishes. Each recording included task-based reading passages and interview-style responses.

The PHQ-9 is widely used for the initial assessment of clinical depressive symptoms (25). The scale comprises 9 items, each scored from 0 to 3, yielding a total score range of 0 to 27. Higher total scores indicate greater severity of depressive symptoms. The original study by Kroenke (25) showed that, with the structured psychiatric interview as the gold standard, a PHQ-9 total score of ≥10 for identifying clinically significant depressive disorders had a sensitivity and specificity of 88% each; this cutoff point is widely used to define moderate or more severe depressive symptoms. In related studies of cancer patients, a PHQ-9 total score ≥ 10 as the cutoff for positive depression (26, 27). In this study, a PHQ-9 score of ≥10 was defined as indicative of depression in cancer patients. When presence of thoughts such as “I might as well be dead” or “hurting myself in some way,” regardless of their persistence, was considered suggestive of depression. The Chinese version of the PHQ-9, standardized and validated for this population, demonstrates an internal consistency coefficient of 0.89 (28).

The GAD-7 provides a quantitative measure of anxiety symptoms through seven items (29). Elevated scores on this scale are closely associated with impairments in multiple functional domains. The original validation study by Spitzer (29) showed that when the total GAD-7 score is ≥10, the sensitivity for identifying generalized anxiety disorder is approximately 89% and the specificity is approximately 82%; this score is recommended as a commonly used cutoff for moderate to severe anxiety symptoms. In this study, anxiety in cancer patients was defined by a GAD-7 score of ≥10. The Chinese version of the scale has been extensively used and shows good reliability, with a Cronbach’ s α coefficient of 0.898 (30).

### 2.2 Voice-text modal depression recognition

The Hidden unit BERT (HuBERT) (31) is a deep learning model specifically designed for self-supervised speech representation learning. Its objective is to learn universal representations directly from unlabeled raw speech data that capture the essential structural properties of speech, including acoustic features, temporal dependencies, and sound unit associations. HuBERT’s ability to model deep acoustic characteristics and capture long-term dependencies enables the direct extraction of depression-related speech features, such as speech rate and fundamental frequency variation, thereby providing a stronger unimodal foundation for multimodal fusion. This model has demonstrated superior performance over traditional approaches in low-resource data settings. In a related study (32), a multimodal approach was proposed that combines a hybrid model combining long short-term memory (LSTM) and convolutional neural networks (CNN) for fine-grained speech analysis with a pre-trained BERT model for text feature extraction. Through decision-level integration, this method achieves 94.30% accuracy and 94.51% F-score in both reading and spontaneous speech depression detection, surpassing current state-of-the-art models.

The depression classification model uses the Transformer-based HuBERT pre-training architecture as the core feature extractor. The model follows a layered design: a convolutional feature extraction layer at the bottom, a 12-layer Transformer encoder in the middle, and a custom classification header at the top. The convolutional layer consists of a 7-layer temporal convolutional network (TCN) that progressively transforms the raw audio waveform into a latent representation, ultimately down sampling the 16kHz audio to a 50Hz frame rate, where each frame corresponding to a 20 ms audio segment. The transformer layer employs 768-dimensional hidden states and 12 attention heads to capture long-range acoustic dependencies via a self-attention mechanism. To adapt the model to depression-specific speech patterns while preserving its general acoustic modeling capacity, we freeze the first 6 layers of Transformer weights and only fine-tune the upper layers. The classification header incorporates a multi-scale feature fusion strategy: the [CLS] labeled representation of the last Transformer layer is first extracted as a global feature; mean-pooled features are also computed for all time-step outputs; finally, a statistical feature layer is added to extract the time-series statistics of depression-related acoustic parameters such as fundamental frequency contour, energy change, and speech rate. During the feature preprocessing stage, we extract three types of acoustic features: rhythmic features, pitch features, and temporal features, and incorporate them into the model via feature splicing. These features are combined in a splicing layer and fed into a two-layer fully connected network with 1024-dimensional and 256-dimensional hidden units, respectively. The network uses the GELU activation function and a dropout rate of 0.3 to prevent overfitting, ultimately outputting a probability distribution for depression.

BERT excels at capturing subtle emotional expressions indicative of mental health states (33). Its bidirectional training structure enables the model to effectively contextualize information from both sides of a marked text segment. In our approach, text is processed by the BERT model to extract semantic features. A binary classifier combined with a majority voting mechanism to output text prediction probabilities. Each text segment contributes one vote, and the final prediction is determined by the majority outcome.

**Figure 1:**
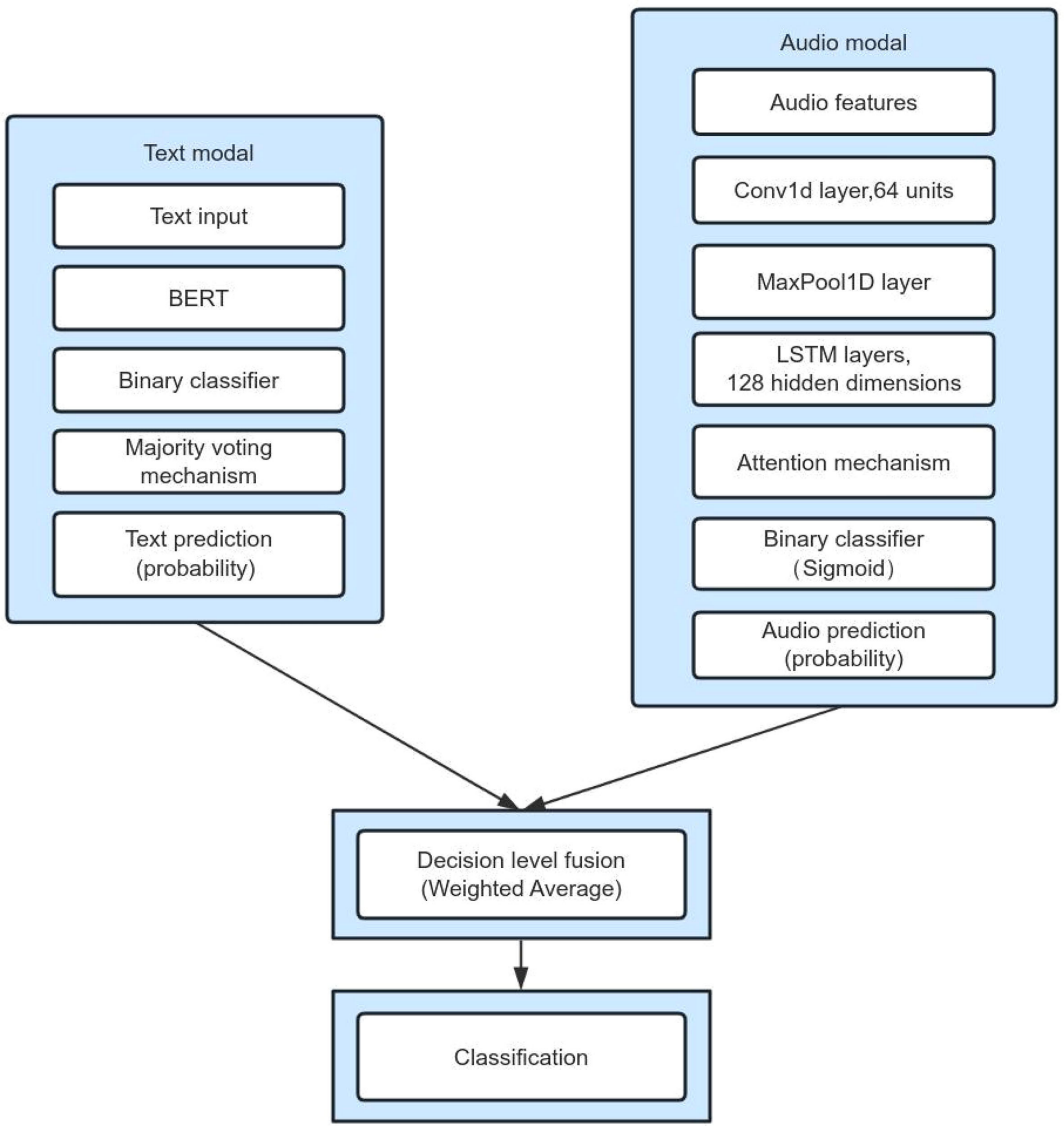
Audio-Text Architecture diagram.

The model features a dual-branch input architecture: a main branch that processes the raw audio waveform, and the auxiliary branch dedicated to the manual features. The training strategy uses progressive unfreezing: only the classification head is trained in the initial phase, the last 4 layers of Transformer are unfrozen in the intermediate phase, and all the upper layers of the network are unfrozen in the final phase. The loss function uses weighted cross-entropy combined with a consistent regularity term to ensure prediction stability across the augmented samples. To address the subjectivity of depression assessment, label smoothing technique is introduced to convert hard labels into soft probability distributions to improve the model generalization ability. In particular, we design temporal attention masking mechanism to strengthen the model’s attention to paused segments of speech, hesitation phenomena, and non-fluent articulatory segments, acoustic events that are highly correlated with depressive states (34, 35). Finally, the speech model and the text model are fused at the decision level using weighted averaging to generate the final output. The model architecture is depicted in **Figure 1**.

We employ HuBERT as the speech encoder because self-supervised pretraining provides hierarchical representations that closely reflect the time–frequency structure of the acoustic signal. The lower Transformer layers predominantly encode relatively stable, task-agnostic acoustic primitives such as short-time spectral envelopes, formant-related energy distributions, voicing-related periodicity, and coarse prosodic modulation. In contrast, higher layers progressively integrate longer-range temporal context and task-specific discriminative information. In psychopathology-oriented speech modeling, available datasets are typically limited in size and heterogeneous in recording conditions, which makes full fine-tuning of the entire encoder prone to overwriting general acoustic priors and to amplifying spurious correlations such as device-dependent bandwidth, room reverberation, or speaker identity leakage. To address this issue, we freeze the first six Transformer blocks, thereby preserving a stable low-level acoustic reference space and concentrating supervised updates in the upper layers. This strategy improves optimization stability and mitigates overfitting, while retaining sufficient flexibility to adapt higher-level temporal dynamics to the downstream task.

In practical training, variable-length utterances must be padded to construct minibatches. Without appropriate masking, attention normalization would distribute probability mass over zero-padded segments that do not correspond to actual speech, which in turn would distort estimates of temporal event density, including pauses, phrase boundaries, and amplitude modulations. To avoid this, we apply an explicit time-domain attention mask that restricts attention computation to valid waveform-derived frames, ensuring that salience is estimated solely from genuine vocal activity. From a psychopathology perspective, many clinically informative cues are expressed through changes in the temporal organization of speech, such as altered pause patterns, slowed or accelerated articulatory pacing, reduced prosodic variability, and irregular energy envelopes, rather than through isolated spectral configurations (36, 37). By combining a frozen low-level HuBERT representation with temporally faithful attention allocation, the model is encouraged to associate textual semantics with behaviorally meaningful acoustic trajectories, while reducing sensitivity to padding artifacts. This design supports a more physically grounded multimodal inference process in contexts where self-report may be affected by stigma and objective behavioral signals provide valuable complementary information.

### 2.3 Facial expression anxiety recognition

**Figure 2:**
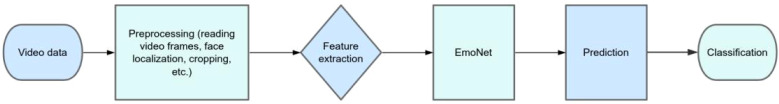
Architecture of the video model.

Theorists claim that emotions represent prototypical responses to situations that have a historical connection to survival. Anxiety symptomatology was assessed using a modified EmoNet (38) architecture (ResNet-50 backbone) with dual-task heads. The model architecture is shown in **Figure 2**. The visual modality model for auxiliary assessment of anxiety and depression is built upon the EmoNet architecture, which integrates Hourglass (HG) modules with convolutional blocks (ConvBlocks) to extract psychologically relevant information from facial expressions.

The network takes as input preprocessed facial images of size 256×256 with three color channels, represented as tensors in CHW format. A Stem block, consisting of a 7×7 convolution with stride 2 followed by batch normalization (BN) and a ReLU activation, is first applied to perform initial feature extraction and spatial downsampling. The relatively large 7×7 kernel provides a broad receptive field at shallow layers, enabling the model to capture global structural relationships among key facial regions, while the stride of 2 reduces the feature-map resolution and computational cost without substantially compromising the overall facial geometry. BN stabilizes activation distributions across mini-batches and facilitates faster convergence with higher learning rates, whereas ReLU introduces nonlinearity, allowing the network to model complex expression patterns and implicitly promoting feature sparsity and regularization.

Following the Stem, three ConvBlocks are stacked sequentially to progressively increase the number of channels to 256, thereby enhancing the representational capacity of the backbone. In this process, the representation transitions from low-level edge and texture features to more semantically meaningful encodings of facial components and expression configurations. An intermediate feature map F at this mid-level stage is preserved for subsequent multi-scale feature fusion.

To construct rich multi-scale representations, two Hourglass modules with a depth of 4 are then stacked on top of the backbone. Each Hourglass module captures both coarse and fine features through repeated downsampling and upsampling pathways. To further improve the consistency and discriminative power of the high-level semantic features, each Hourglass module is followed by a ConvBlock and a 1×1 convolution–BN sequence, which serve to aggregate multi-scale information and produce high-level feature maps encoding both global facial structure and local details.

On top of these high-level features, an attention mechanism is introduced. The network first predicts a set of 68-channel keypoint heatmaps, which are designed to correspond to salient facial landmarks. These heatmaps are passed through a 1×1 convolution followed by a Sigmoid activation to generate a spatial attention map. This attention map is applied to the high-level feature maps in a residual manner, selectively emphasizing regions that are particularly informative for emotional inference—such as periocular, eyebrow, and perioral areas—while suppressing less relevant regions.

Finally, the attention-refined high-level features from the Hourglass pathway are concatenated with the intermediate feature map F from the backbone, thereby integrating information across different spatial scales and network depths. A subsequent 1×1 convolution reduces the channel dimensionality to 256, yielding a compact yet expressive fused representation. This fused representation is then passed through four ConvBlocks with max-pooling to derive progressively higher-level, more abstract features, followed by a 4×4 average pooling layer that produces a global feature vector. A fully connected layer maps this vector to a 128-dimensional shared feature representation, which is ultimately fed into the output layer to generate the final prediction of anxiety versus non-anxiety.

### 2.4 Multimodal fusion strategy

**Figure 3:**
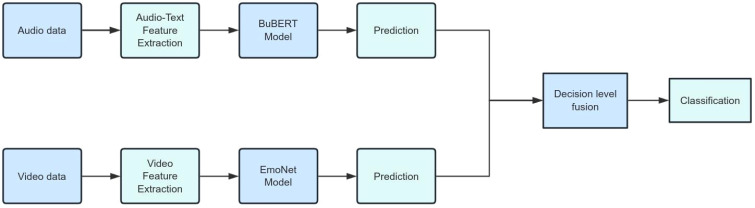
Decision-level fusion for multimodal depression-anxiety detection.

In this study, we fused modalities at the decision level. Decision-level weighted averaging is a commonly used fusion method in healthcare scenarios, which involves combining the outputs of multiple independent models, each dealing with a different data modality, to make a final decision, as shown in **Figure 3**. This approach is very effective when dealing with different feature sets and can capitalize on the strengths of each model, thus potentially enabling more robust and accurate predictions (39). Compared to other fusion paradigms, including early fusion and intermediate fusion architectures based on cross-modal attention or collaborative attention transformers (40), decision−level averaging has relatively modest requirements for temporal alignment across modalities and is less demanding in terms of dataset size and computational resources (41, 42). This is particularly relevant in our setting, where the available labeled dataset is moderate in size and deployment is intended on standard hospital hardware without specialized accelerators. For these reasons, we used decision−level weighted averaging as the primary multimodal fusion strategy in this work.

### 2.5 Evaluation metrics

In this study, the analysis and mitigation of class imbalance primarily involve the selection of appropriate evaluation metrics. The dataset exhibits significant class imbalance; under such conditions, accuracy alone cannot provide a reliable assessment of model performance. We therefore adopt a comprehensive set of evaluation metrics including accuracy, precision, recall, F1 score and AUC to systematically evaluate model performance. These metrics are more robust to imbalanced data distributions and can better reflect the actual screening ability of the model in clinical practice. We also acknowledge as a limitation that the current training objective uses standard cross-entropy without explicit class reweighting/sampling, and we note this as future work (e.g., class-weighted loss, focal loss, or weighted sampling) to further strengthen minority-class sensitivity.

## 3 Results

### 3.1 Distribution of psychological assessment scale scores

A total of 1,089 samples were collected, of which 40 were deleted due to incomplete information resulting from technical issues.

The PHQ-9 score ranges from 0 to 27 and is used to assess the severity of depression. Its distribution in this study was characterized as follows: A total of 868 cases were defined by <10 points (defined as depression-negative); a total of 181 cases were defined by ≥10 points (defined as depression-positive); a total of 1049 samples were included. In terms of distribution, 17.25% of patients were depression-positive.

The GAD-7 score ranges from 0 to 21 and is used to assess anxiety severity. Its distribution in this study was characterized as follows: a total of 898 cases were defined by a score of <10 (defined as anxiety-negative); a total of 151 cases were defined by a score of ≥10 (defined as anxiety-positive); a total of 1049 samples were included. In terms of distribution, 14.39% of patients were anxiety-positive.

### 3.2 Unimodal model recognition performance

This study focuses on multimodal mental state recognition, and evaluates the efficacy of different modalities recognizing depression and anxiety by comparing the performance of unimodal model and decision-level fusion model on the whole dataset, with the key metrics of Area Under the Curve (AUC), Accuracy, F1-score, Precision and Recall. The results are summarized as follows. The performance metrics of the models are shown in **Table 1**.

**Table 1:** Comparison of unimodal and multimodal performance across the entire database.

Model/Fusion method	AUC	Accuracy	F-score	Precision	recall
Audio model	0.79	0.75	0.75	0.76	0.74
Text model	0.77	0.71	0.74	0.72	0.76
Audio-Text model	0.81	0.76	0.77	0.78	0.76
Video model	0.77	0.72	0.72	0.73	0.71
Decision-level Fusion (depression)	0.89	0.83	0.85	0.85	0.84
Decision-level Fusion (anxiety)	0.79	0.74	0.74	0.75	0.73

#### 3.2.1 Audio model

The audio model performs best in all unimodal modes, with leading core metrics: an AUC of 0.79, (highest among unimodal modes), an accuracy of 0.75, an F1-score of 0.75, a precision of 0.76, and a recall of 0.74. This result aligns with the findings of Daly (32), which indicates that rhythmic, spectral features in audio signals are a key source of information for mental state recognition. The high AUC of the audio modality in the present study further validates this view, suggesting that audio features such as intonation and speech rate are strongly discriminative in the discrimination of depressive mood and are the core information carriers in the unimodal context.

#### 3.2.2 Text model

The text model demonstrates robust performance in unimodal settings, with core metrics as follows: an AUC of 0.77, an accuracy of 0.71, an F-score of 0.74, a precision of 0.72, and a recall of 0.76. The higher recall is closely related to the informational properties of the text modality. The semantic tendencies and emotional vocabulary embedded in the text can directly reflect mental states, making it more adept at capturing “potentially positive samples” and reducing missed detections.

#### 3.2.3 Video model

The overall performance of the video model is comparable to that of the text model, with core metrics as follows: an AUC of 0.77, an accuracy of 0.72, an F-score of 0.72, a precision of 0.73, and a recall of 0.71. Its accuracy is slightly higher than that of the text model. Changes in facial expressions are highly specific and can effectively reduce misdiagnosis. Consistent with Chen’s (16) findings that “facial features have high specificity”, these results further confirm the value of video modality in improving recognition accuracy.

### 3.3 Decision-level fusion model recognition performance

#### 3.3.1 Audio-text model

To validate the multimodal synergy effect, this study constructed an Audio-Text model, whose performance was significantly improved over the single-modal model: an AUC of 0.81, an accuracy of 0.76, a precision of 0.78, and a recall of 0.76. This result indicates that the acoustic features of the audio modality and the semantic features of the text modality are complementary. The audio modality can capture non-verbal emotional signals, and the text modality can provide verbal-level emotional expressions. and The combination of the two can enhance the reliability of emotional disorder detection and lay the foundation for subsequent more complex fusion strategies.

#### 3.3.2 Audio-text-video model

The overall performance of the decision-level fusion model is better than that of the text, video and audio-text combination models. The Audio-Text-Video model outperforms other models in detecting depression in all indicators: an AUC of 0.89, an accuracy of 0.83, a precision of 0.85, and a recall of 0.84. This result suggests that the decision level fusion model can effectively integrate the complementary information related to depression in multimodal modalities, reduce the information bias of the unimodal model, and significantly improve the accuracy and stability of depression detection. The results indicate that the decision layer fusion model can effectively integrate the complementary information related to depression in multimodality, reduce the information bias of the unimodal model, significantly improve the accuracy and stability of depression detection, and provide key technical support for the development of automated depression screening tools. For anxiety, the AUC is 0.79, the accuracy is 0.74, the F1 score is 0.74, the precision is 0.75, and the recall is 0.73. This validates the effectiveness of decision-level fusion in anxiety recognition.

## 4 Discussion

### 4.1 Principal findings and clinical interpretation

In this monocentric cohort of hospitalized Chinese cancer patients, we developed and internally validated a multimodal artificial intelligence system that screens for anxiety and depression based on speech and facial expressions. Using PHQ-9 and GAD-7 cut-offs as reference standards, the multimodal fusion model demonstrated good discrimination, particularly for depression, and clearly outperformed unimodal models.

Importantly, we explicitly position the model as a clinical screening aid rather than a diagnostic tool. Its intended role is to identify patients at increased risk of significant distress and to prompt further evaluation by mental health professionals. In this sense, the model is conceptually analogous to questionnaire-based screening, but it relies on behavioral signals that may be less affected by social desirability and stigma-related under-reporting.

In this study, using the GAD-7 and PHQ-9 as assessment criteria, the authors found that 17.25% of participants tested positive for depression and 14.39% tested positive for anxiety. Due to the stereotypes, prejudice and discrimination against severe mental disorders in the outside world, many patients will perceive and identify with these negative social attitudes, internalize them into themselves, and have mental illness-related stigma (43). Therefore, many patients choose low or even zero scores to hide their true feelings in doctor-patient communication. This phenomenon may be an important reason why the detection rate of anxiety and depression based on GAD-7 and PHQ-9 in China is significantly lower than that in other countries. In addition, some patients with more severe symptoms and poorer communication skills were excluded because they were unable to complete the assessment process successfully; consequently, the patients ultimately included in the analysis were predominantly those with relatively stable conditions, which may also have led to an underestimation of the actual levels of anxiety and depression among cancer patients. In this study, the multimodal fusion model is expected to have a depression recognition F1 value of 0.85 and an anxiety recognition F1 value of 0.74, which are significantly better than the unimodal model. These results suggest that speech, language, and facial expression recognition have important diagnostic value in the classification of depression and anxiety in cancer patients (44, 45).

### 4.2 Methodological considerations and fusion strategy

Our choice of decision-level fusion has important methodological implications. Because each modality is processed independently and only combined at the probability level, the current model effectively assumes that the contribution of each channel is additive and conditionally independent given the outcome. This simplifies modeling but also constrains the types of cross-modal patterns that can be learned (46).

Clinically, many psychologically relevant signals may lie precisely in the inconsistency between modalities. In a decision-level framework, such patterns are captured only indirectly, via the separate probabilities from each modality, without explicitly modeling their interaction structure. More expressive fusion schemes, such as feature-level concatenation, cross-modal attention, or shared latent spaces, are specifically designed to learn these dependencies (47). For instance, a cross-modal attention module could attend to facial regions that change their diagnostic value conditional on the semantic content or prosodic contour of the utterance. However, these architectures are more parameter-rich and usually require larger, well-aligned multimodal datasets, as well as greater computational resources and engineering complexity.

Future work in psycho-oncology should therefore move beyond a single fusion strategy and formally compare different fusion levels under controlled conditions. Possible designs include: (1) training feature-level and cross-modal models on the same dataset and reporting not only discrimination but also calibration and robustness across subgroups; and (2) conducting ablation studies that quantify how much of the performance gain comes from capturing cross-modal inconsistencies versus simply aggregating stronger unimodal signals. Such studies would help clarify whether the additional complexity of advanced fusion architectures translates into clinically meaningful improvements in screening performance and justifies the cost of deployment in real-world oncology settings.

### 4.3 Relation to previous work in digital psycho-oncology

Traditional methods, while valuable, are often time-consuming and subject to both patient and clinician bias (13, 14). The present system, by contrast, offers a non-invasive, scalable, and objective means of assessment that can be administered repeatedly with minimal burden on patients or clinical staff (48).

While the system is designed for screening and not diagnosis, its outputs could influence clinical decision-making. It is therefore essential that such tools are transparent in their limitations and uncertainties. Moreover, the issue of data privacy and security cannot be overlooked. Audio and video data are highly sensitive, and robust measures must be in place to ensure anonymization, secure transmission, and ethical storage. Compliance with regional data protection regulations is mandatory for any clinical implementation.

Finally, the long-term utility of such a system depends on its integration into clinical workflows. It should not replace human judgment but rather augment it, providing clinicians with additional, quantifiable insights. Pilot implementation studies are needed to assess usability, acceptability, and impact on patient outcomes. If successful, this approach could be extended to other populations, such as patients with chronic diseases or survivors in long-term follow-up, where mental health monitoring is equally critical.

In summary, while the current model shows promising accuracy and robustness, its true value will be determined by its ability to integrate seamlessly into care pathways, its generalizability across diverse populations, and its capacity to evolve with advancing AI methodologies and growing clinical datasets.

## 5 Limitations

The study was limited to a single-center investigation with potential selection bias. This is a single-center study conducted at a tertiary hospital in Wuhan, China; as such, it is population-specific, and the model’s performance may not be generalizable to other regions of China or to populations of different ethnic or cultural backgrounds. A study found that white patients had higher accuracy in recognizing emotions, while black patients had the worst accuracy. De Hond (49) found an underestimation of adverse psychological risk in female and black patients. Furthermore, the study did not account for the impact of the stage of cancer treatment on model performance, which further limits the generalizability of the findings. Potential selection bias constitutes another notable limitation. Patients with more severe symptoms or poorer communication skills may have been unable to complete the recording tasks and were thus excluded from the study. The prevalence of anxiety and depression in our sample is lower than global meta-analytic estimates, suggesting our model was trained on a relatively healthier subset of the target population, which may affect the external validity of the results.

The model lacks sufficient validation and benchmarking. It was evaluated only on internal test data from the same cohort, and external validation on an independent, geographically distinct dataset is essential before any real-world deployment. Furthermore, we did not benchmark model performance against the clinical judgment of oncologists, nurses, or psychiatrists, leaving the model’s added value over existing clinical workflows unquantified. Current artificial intelligence models for mental health assessment, including ours, face multiple conceptual and practical limitations. Most rely on questionnaire-derived cut-offs rather than structured clinical diagnoses; while such questionnaires are valid and convenient, they serve as imperfect surrogates for major depressive disorder and generalized anxiety disorder. Model training often uses small, single-center convenience samples, increasing overfitting risk and limiting generalizability (50). The model has not undergone prospective, real-world testing and has not been deployed in a live clinical environment. Factors such as background noise, variable recording conditions, patient non-cooperation, and integration with electronic health records were not assessed, which may affect its performance in practical clinical settings.

Another limitation concerns the way the facial-expression branch operationalizes anxiety. In its current formulation, the visual network adopts a multi-task learning framework to simultaneously predict multiple basic emotion categories alongside the binary anxiety versus non-anxiety classification label, with the anxiety score integrated into the multimodal fusion framework derived directly from the sigmoid output of the dedicated anxiety binary classification head. Probabilities associated with basic emotions function primarily as a form of auxiliary supervision, and are not explicitly integrated into a continuous anxiety index through a transparent mathematical mapping process, including predefined or data-driven weighting of emotion categories such as fear and sadness. Future work should further explore more explicit and clinically meaningful mapping approaches to address this gap. This line of inquiry may include defining and validating theory-based or data-driven weighting schemas to aggregate basic emotion probabilities into a continuous facial anxiety activation score, as well as systematically analyzing how fluctuations within individual emotional channels, including fear, neutral expression, and suppressed positive affect, shape the model’s predicted anxiety risk. Such investigations would not only enhance the transparency of the model for clinical end users, but also help clarify whether basic emotion outputs offer incremental utility beyond simple binary anxiety prediction within the psycho-oncology context.

Our study did not compute confidence intervals for performance metrics, constraining interpretation of result stability and uncertainty. Model interpretability also remains challenging, as black-box algorithms may not be trusted by clinicians without explanations of prediction mechanisms.

Future work should prioritize larger, more diverse datasets, multi-site external validation and rigorous reporting of uncertainty and calibration. Integrating explainable AI techniques and user-centered design will also be essential to ensure model outputs are interpretable and actionable for clinicians and patients (51).

## 6 Conclusion

In this paper, we proposed a multimodal fusion method that uses an optimized HuBERT architecture for speech depression recognition and multi-task CNN for facial anxiety recognition from facial expressions. This approach, combined with decision-level weighted averaging, significantly improved the assessment accuracy. The method is specifically tailored to the unique vocal and facial characteristics of cancer patients. Within its current limitations, the system should be understood as a complementary screening tool rather than a diagnostic device. It may help clinicians identify patients who warrant more thorough psychological assessment, particularly in settings where time constraints, stigma, and patient fatigue limit the use of traditional questionnaires and interviews.

Future research should focus on external validation across multiple centers and cultures, more sophisticated fusion strategies that capture inter−modal dependencies, and prospective studies that assess clinical utility, interpretability, and ethical implications. Only through such work can multimodal artificial intelligence tools be responsibly integrated into psycho−oncology care and contribute to earlier detection and better management of anxiety and depression in patients with cancer.

## Data Availability

The datasets presented in this article are not readily available due to confidentiality requirements and ethical constraints. Requests to access the datasets should be directed to Na Xu, na.xu123@outlook.com.
